# Two-Sided Matching for mentor-mentee allocations—Algorithms and manipulation strategies

**DOI:** 10.1371/journal.pone.0213323

**Published:** 2019-03-12

**Authors:** Christian Haas, Margeret Hall

**Affiliations:** College of Information Science and Technology, University of Nebraska at Omaha, Omaha, NE, United States of America; Virginia Commonwealth University, United States of America

## Abstract

In scenarios where allocations are determined by participant’s preferences, Two-Sided Matching is a well-established approach with applications in College Admissions, School Choice, and Mentor-Mentee matching problems. In such a context, participants in the matching have preferences with whom they want to be matched with. This article studies two important concepts in Two-Sided Matching: multiple objectives when finding a solution, and manipulation of preferences by participants. We use real data sets from a Mentor-Mentee program for the evaluation to provide insight on realistic effects and implications of the two concepts. In the first part of the article, we consider the quality of solutions found by different algorithms using a variety of solution criteria. Most current algorithms focus on one criterion (number of participants matched), while not directly taking into account additional objectives. Hence, we evaluate different algorithms, including multi-objective heuristics, and the resulting trade-offs. The evaluation shows that existing algorithms for the considered problem sizes perform similarly well and close to the optimal solution, yet multi-objective heuristics provide the additional benefit of yielding solutions with better quality on multiple criteria. In the second part, we consider the effects of different types of preference manipulation on the participants and the overall solution. Preference manipulation is a concept that is well established in theory, yet its practical effects on the participants and the solution quality are less clear. Hence, we evaluate the effects of three manipulation strategies on the participants and the overall solution quality, and investigate if the effects depend on the used solution algorithm as well.

## Introduction

Committee memberships, group assignments, or internship allocations: There are may situations where decisions need to be made based on the participants’ preferences. Two-Sided Matching is a scientifically grounded approach to determine such allocations when participants provide their preferences and allocations should be determined without involving monetary transactions. While finding such an allocation (or solution) to the problem at hand is often an NP-hard optimization problem [1], different approaches in Two-Sided Matching allow for efficiently calculating solutions with desirable properties [2].

Over the years, several different approaches have been developed to find these preference-based allocations with good properties. In the general setting, many approaches focus on finding a stable solution (no participant has an incentive to deviate from it) that matches as many participants as possible [3]. These approaches are commonly either heuristics or approximation algorithms with a guaranteed worst-case performance. Multi-objective heuristics have been proposed that also take into account secondary goals such as finding solutions that are as beneficial as possible for the participants by matching them to (or close to) their preferred solutions [4]. However, it is hard to estimate the practical performance of these algorithms without applying them to realistic scenarios and preferences.

Additionally, a potentially major caveat in Two-Sided Matching is that some participants will have an incentive to provide manipulated, altered preferences as it could improve their outcome. There has been some theoretical and experimental work in this area that provides initial results (e.g., [5], [6]), yet it is less clear how beneficial or severe such potential manipulation can be for realistic preferences, and how it affects the overall solution for Two-Sided Matching.

In this article, we address both of the previous aspects by providing a systematic comparison of state-of-the-art algorithms in Two-Sided Matching with respect to multiple quality metrics, and by analyzing the effects of preference manipulation on the outcome. Building on a previous study [7], we use three realistic sets of preferences stemming from a mentor-mentee program at our university, to conduct the analysis. Through systematic simulation-based evaluation, we compare different algorithms in their ability to create good solutions, and consider different manipulation strategies.

We consider two main research questions in this article:

1. **Research Question 1**: For the considered realistic data sets, what is the performance of different solution algorithms relative to the optimal solution?
2. **Research Question 2**: How does preference manipulation affect the participants’ outcome and the overall solution quality?

Answering these research questions will benefit the Two-Sided Matching research and community by providing guidance on which algorithms to select for similar settings. In addition, it will provide valuable insights into the severity and prevalence of potential preference manipulation. Together, the evaluation in this article will foster the use of Two-Sided Matching for calculating allocations in preference-based settings.

The article is structures as follows. In Section 1 we provide the necessary foundations of Two-Sided Matching, including the specification of commonly considered scenarios. We discuss related work relevant to this article in Section 2, and specify the methodology and setup of this simulation-based study in Section 3. In Sections 4 and 5, we evaluate the performance of the various algorithms and the effects of preference manipulation, respectively. Finally, we discuss the results in Section 6 and conclude our study with a summary and outlook on future work in Section 7.

## Two-Sided Matching foundations

We consider the matching of a set of mentors, *X*, and mentees, *Y*, in a university advising program that encourages women to pursue a career in computing. The task at hand is to find a *match* of mentors with potential mentees, 〈*X*, *Y*〉 consisting of pairs of individuals 〈*x*, *y*〉 where *x* ∈ *X* and *y* ∈ *Y*, while aiming to ensure that both sides are as happy with the matching as possible. As the number of mentors and mentees does not have to be equal, a match does not guarantee that each mentor or mentee will be matched. However, as in our setting the number of mentors is higher than the number of mentees, our goal is to find a mentor for each mentee.

Both mentors and mentees have *preferences* $P_{i}=(P_{i,j_{1}},\ldots ,P_{i,j_{n}})$, *j* ∈ *Y* and *i* ∈ *X*, or *i* ∈ *Y* and *j* ∈ *X* with whom they want to be matched. The preferences are represented by ordinal ranks, where *P*_*i*,*j*_ denotes the preference rank that user *i* has towards user *j*. The most preferred option (match) has rank 1, the second-most preferred match rank 2, and so on. The preferences represent transitive priority structures ≿, where each user of the opposite side is ranked according to its priority. The asymmetric part ≻_*i*_ indicates a strict priority, whereas the symmetric part indicates an indifference. For example, *j*_1_ ≻ *j*_2_ means *j*_1_ is preferred over *j*_2_, whereas *j*_1_ ∼ *j*_2_ means the user is indifferent between *j*_1_ and *j*_2_. The preference towards being unmatched is defined as *P*_*i*,∅_. If user *j* appears in user *i*’s preference list, *j* is said to be **acceptable** for *i*. Similarly, unacceptable users do not appear in the preference list. If one or more users *j* are unacceptable for user *i*, the respective preferences are said to be **incomplete** as these users are missing in the preference ranking. This indicates that user *i* wants to remain unmatched rather than being matched to users *j*. A preference profile is **strict** if ∀*j* ∈ *X* (*j* ∈ *Y*), ≿_*j*_ is asymmetric. If *j* ∼ *k* for some users *j* and *k*, then the preference profile is said to have **indifferences**, or ties.

In our case, we allow for preferences that are both incomplete and include ties. Mentors are allowed to place restrictions on the specific mentees that they are willing to supervise (e.g., some mentors only want to be matched with undergrad students). Similarly, in our setting mentees provide the names of up to 5 mentors that they would prefer. While this restriction seems arbitrary, we decided to limit the number of mentors that each mentee has to rank to reduce the time (and complexity) for the mentees to form a ranking out of a large pool of mentors. Due to these restrictions, preferences on both sides are *incomplete*. Furthermore, mentees are allowed to be indifferent between their listed options. For example, they might state that some mentors are equally preferred, while the remaining mentors are less preferred. Mentors do no provide a further preference ranking, i.e., are by default indifferent between all allowed options (i.e., allowed mentees after the previous restrictions).

### Finding optimal solutions

Based on these stated preferences, Two-Sided Matching calculates a match 〈*X*, *Y*〉 where mentors are matched with mentees (or not matched at all). To evaluate the quality of a matching between mentors and mentees, we use following standard criteria to consider the solution quality: stability, the number of matched pairs, and the average welfare.

**Stability** is the core requirement of practically all approaches for Two-Sided Matching. In a stable solution, no participant can be better off (be matched with a more preferred mentor/mentee) by switching their allocated partner with another mentor/mentee pair. Proposed mentor-mentee matches may fail in the sense of become undone after having been proposed, because some participants see better opportunities. Specifically, a match 〈*X*, *Y*〉 containing at least one pair of matched individuals 〈*x*_1_, *y*_1_〉 and 〈*x*_2_, *y*_2_〉, *x*_1_, *x*_2_ ∈ *X* and *y*_1_, *y*_2_ ∈ *Y*, where *x*_1_ prefers *y*_2_ to *y*_1_ as a partner and *y*_2_ prefers *x*_1_ to *x*_2_ as a partner is said to be *unstable*. If the unhappy participants, here *x*_1_ and *y*_2_, are aware of this, they would break their proposed mentor relationships and form a new pair. Stability is important as otherwise even one unstable pair can lead to chaotic unraveling [8]. Note that stability is not further described as solution property as all algorithms considered in this paper yield stable solutions, thus removing the requirement to compare the stability of the different approaches.

The second criteria is the **cardinality** (number of matched pairs) of the match, i.e., the number of pairs 〈*x*, *y*〉 where *x* is a mentor and *y* is a mentee. This might seem obvious, yet finding a solution with the maximum number of matched pairs is itself an NP-hard problem [1]. In our context, we aim to provide a mentor-mentee match for as many mentees as possible as it is not guaranteed that a stable solution exists where all mentees are matched.

As a third criteria, the average preference rank that each participant is matched with is considered as **Welfare** of the solution (see e.g. [9] for a similar definition of an egalitarian metric). That is, for each matched participant we sum up the preference rank *P*_*ij*_ of their respective match and calculate the mean matched preference rank:

$$\begin{array}{c} Welfare=\sum_{(x,y)\in MatchedPairs}P_{x}y+P_{y}x \end{array}$$ (1)

For example, given that the highest (most preferred) rank is 1, a welfare score of 2 would mean that participants are, on average, matched with their second most preferred choice. While welfare is an important criteria for the solution, it is not commonly considered by many approximation algorithms or heuristics in the general case of incomplete preferences with ties, due to the NP-hardness of the problem. However, it can be easily included in evolutionary algorithms as additional objective (see e.g., [4]). Also, note that the Welfare criterion only relies on the preference ranks for calculation and thus does not consider non-linear utilities for different options. In other words, the preference difference between choice 1 and 2 is the same as the preference difference between ranks 2 and 3. This follows the main literature in Two-Sided Matching, yet could be extended to include cardinal utilities (e.g., normally or exponentially distributed utilities). As this would require the consideration of weighted preferences and extensions of the stability concept (and thus falls outside the main Two-Sided Matching literature), we do not consider cardinal utilities here.

Finally, **Equality** considers if both sides of participants are treated equally. For each matched pair of mentor and mentee, the rank of the respective matched partner is calculated. The equality score for a pair is defined as the difference of these two ranks. A solution is considered to be more equal if both mentor and mentee are close in the respective other preference ranking. A solution that is completely equal would have score of 0 in this case.

Given these solution quality criteria, we aim to find a stable solution that matches as many mentees as possible, while at the same time matching each participant as close to their most preferred choice as possible. Several algorithms have been suggested for this case, and they are described in the next section.

### Preference manipulation

After defining the fundamental concepts of Two-Sided Matching, we can now consider preference manipulation. Ideally, we want to find solutions which are simultaneously stable and yield optimal values for the number of matched pairs, welfare, and equality, while at the same time being immune to manipulation from participants. Unfortunately, Mechanism Design tells us that only a certain combination of desired solution properties can be achieved in Two-Sided Matching, and impossibility theorems provide valuable guidance in this case (see e.g. [10] for an overview).

The question if participants submit their true or manipulated preferences is of particular interest. From the viewpoint of the overall system, having a mechanism which is immune against manipulation (incentive compatible) is important for several reasons. On one hand, such a mechanisms guarantees that stable solutions calculated with the submitted preferences will also be stable under the true preferences, i.e., we can be sure that the calculated solution will be acceptable for all participants. On the other hand, participants know that it is in their best interest to actually submit their true preferences, avoiding the need to decide on potentially complex strategies. However, considering that stability is a fundamental requirement for Two-Sided Matching solutions, the question is whether stability and incentive compatibility can be achieved together. The fundamental result considering incentive compatibility in Two-Sided Matching was developed by [3] and summarized in [11]:

**Theorem 1** ([3]) *No stable matching mechanism exists for which stating the true preferences is a dominant strategy for every participant*.

From a preference manipulation perspective, this result has serious implications on the design of a Two-Sided Matching mechanism. As stability is commonly (and empirically) considered the most important property (see e.g. [2]), incentive compatibility needs to be sacrificed if stability is to be guaranteed, which is the case for practically all current algorithms. From a preference perspective, this result implies that some participants *i* have an incentive to submit preference profiles $P_{i}^{*}$ to the mechanism, where $P_{i}^{*}\neq P_{i}$.

Although this seems to be a rather negative result, the implications of the impossibility of incentive compatibility for all participants are less clear. As [12] noted: “However the existing theoretical results do not generally allow us to address the considerable demand for practical advice about how to participate in such markets, once they are established. It is difficult to advise participants in markets that use stable matching mechanisms when to behave straightforwardly (i.e. in a way that reveals their true preferences) and when there might be opportunities to behave strategically, and if so, how. This also suggests that there are some gaps in our understanding of why stable matching mechanisms work so well in practice” [12, p.21]. For example, as one of the few results in the literature, [12] find that only a small number of participants have incentives to reveal altered preference rankings. Additionally, [6] show that there can be non-strategy-proof matching mechanisms which are NP-hard to manipulate, i.e., finding a successful manipulation strategy can be a hard problem.

## Related work

In their seminal paper on Two-Sided Matching, [13] introduced the Deferred Acceptance (DA) algorithm as a method to find a stable solution in case of complete preferences without ties. While the DA guarantees to find a stable solution efficiently for a special case of Two-Sided Matching, it sparked the study of more general scenarios and the development of further algorithms. As such, related work in Two-Sided Matching can be broadly categorized into solutions for different preference properties and solution quality criteria, and the analysis of preference manipulation strategies.

Regarding preference properties and solution quality, we have to distinguish between complete and incomplete preferences as well as the introduction of ties (indifferences). On one hand, the DA can be used in all scenarios to compute a stable solution. On the other hand, including additional solution criteria leads to considerably more complex problems. For example, [8, 14] showed that the number of stable solutions for a given preference set can be large, sometimes even exponential. With the exception of complete preferences without ties, where there are polynomial-time algorithms to compute the welfare-best [15] and approximately fairness-best solutions [9], finding the welfare-best or fairness-best stable solution is generally an NP-hard problem and sometimes even hard to approximate [16]. For the most general case of incomplete preferences and allowed indifferences, the common goal is to find stable solutions that match as many participants as possible. For this specific case, approximation algorithms have been developed that provide lower-bound quality guarantees for the solutions [17–19]. In addition, other approaches that aim to increase the solution quality are heuristics such as Genetic Algorithms with multi-objective target functions [4, 20, 21]. While heuristics do not provide lower bound quality guarantees, they have been shown to work well on average and offer the flexibility to optimize multiple solution criteria simultaneously. In another approach, Erdil and Ergin [22] introduced an extension to the DA that can cope with ties in preferences. Their algorithm tries to find potential Pareto-improvement cycles in a given solution which might improve the overall quality of the solution.

Based on the practical relevance of potential manipulation effects, such as potentially increasing or decreasing one’s chances of getting into a particular school/college/hospital, previous research on preference manipulation has concentrated on two main aspects. First, the theoretical existence and properties of manipulation. Second, the effects of manipulation in selected settings, e.g., the well-understood Deferred Acceptance Algorithm [13] or for many-to-one matching scenarios.

Considering the existence of preference manipulation, [3] showed that there can be no incentive-compatible solution algorithm in standard Two-Sided Matching scenarios that always yields a stable solution. Analyzing its strategic properties, the DA is strategy-proof for the proposing side, yet not strategy-proof for the accepting side [11]. Moreover, not putting its most preferred alternative first is a dominated strategy for the participants of the accepting side [11, p. 105]. [5] show that (again in the case of strict preferences) for any tie breaking rule, there is no mechanism that is strategy-proof and dominates the DA. [23] consider manipulation in the DA and show that all weakly beneficial group manipulation strategies of accepting participants are beneficial for all other accepting participants and harmful for all proposing participants. Furthermore, this is true if participants from the accepting side apply a truncation of preferences. Studying the prevalence of manipulation in experimental settings, [24] show that truncation of preferences for the accepting side in a DA is applied only rarely. Through structured laboratory experiments, [25] analyze the prevalence of preference truncation on a number of different factors. Knowing the theoretically best truncation strategy in a given scenario, they show that the existence of theoretically profitable truncation does not increase the chances that participants manipulate their preferences. However, they show that the risk of being worse off due to truncation (e.g., when truncating too much) leads to a decrease in observed preference truncation.

[26] study optimal degrees of truncation if one side of participants manipulate their preferences in the standard DA setting. They find that the optimal truncation degree can be substantial, especially for systems with many participants, and that an equilibrium exists where everyone manipulates. In addition, they show that when preferences are correlated, the optimal truncation degree is lower than for uncorrelated preferences.

In many-to-one settings, several studies looked at the effects of manipulation in the respective markets. Under certain conditions, the percentage of participants that can successfully manipulate their preferences in a student-optimal stable matching converges to zero for large markets [27, 28]. [5] discuss the effect of strategy-proofness on efficiency (measured in average rankings) in the school-choice problem using date from New York and Boston school districts. Furthermore, [29] studies manipulation strategies in the school choice problems. In particular, [29] considers the option for schools to manipulate their submitted capacity (i.e., offering less capacity than available), and the possibility to pre-arrange matches in which case the involved student does not participate in the actual matching procedure. Whereas some of the studied mechanisms are immune to capacity manipulation, [29] shows that all studied mechanisms are not immune to pre-arranged matches. [30] suggest a method to compare how manipulable matching algorithms in the School Choice setting are, and show that changes made to the School Choice algorithms in Chicago and England are justified as they decreased the potential manipulability of the algorithms. In addition, [24] show that when the DA is applied in a practical setting, such as National Resident Matching Program, only about half of the resulting outcomes were stable. In addition, preference manipulation occurs much more frequently on the side of participants applying for a certain position or spot, as compared to the side of the offering participants (hospitals, schools, etc.).

## Methodology and approach

### Solution quality

Given the preferences and solution evaluation criteria introduced in the previous chapter, we compare a set of different algorithms in their ability to find a good solution for this NP-hard problem. As a baseline, we use the previously introduced Deferred Acceptance (DA) algorithm. Having been developed to find a stable solution in case of complete preferences without ties, it does not guarantee that the maximum number of mentor/mentee pairs are found in case of incomplete preferences with ties. However, by breaking the ties (even in an arbitrary fashion), the DA can calculate a stable solution based on this tie breaking, which produces a stable solution under the original preferences (see, e.g., [1]). We use this approach of arbitrary tie breaking for the DA in the following evaluation. In addition, as solutions generated by DA under strict and complete preferences yield an optimal solution for one side and a pessimal solution for the other side, our DA implementation uses a mentee-proposing version as we wanted the matching results to be favorable for mentees. Besides the DA, we compare two general sets of approaches that aim to find a solution with as many matched pairs as possible: Approximation algorithms and heuristics.

### Approximation algorithms

Approximation algorithms provide a guaranteed performance with respect to the number of matched pairs. In other words, given the actual optimal solution (the one with the highest number of matched pairs), the calculated solutions are within a certain boundary to this optimal solution. We consider following approaches that have been proposed in the previous years (for a description of the underlying pseudocode for each algorithm we refer to the respective publication):

- *Shift*: [31] describe an approximation algorithm for this case, in the following abbreviated as *Shift*. For certain preference structures, this algorithm provides non-trivial quality bounds for finding the stable match of maximum size. Shift operates through breaking indifferences in a systematic manner and applying the DA on the resulting set of strict preferences. In particular, if indifferences occur on both sides of the market, Shift guarantees non-trivial quality bounds if the length of indifferences is at most 2.
- *Király*: [18] presents an algorithm with a 5/3 approximation ratio in the general stable matching case, and a 3/2 approximation ratio when ties are only allowed on one side.
- *McDermid*: [17] presents an algorithm that improves upon the algorithm by [18] by providing a 3/2 approximation ratio, which is the best known approximation ratio for the general case without restrictions on tie lengths.
- *GSModified*: [19] presents another algorithm with the same 3/2 approximation ratio as the algorithm by [17], yet with an additional improvement in runtime.

### Heuristics

In contrast to approximation algorithms, heuristics do not provide a guaranteed solution quality. However, it is less clear how the algorithms perform on average. Previous research considered different types of heuristics which are described below. We focus on the Genetic Algorithm heuristic in the subsequent evaluation.

- *LocalSearchSMTI*: [32] present local search heuristics to solve the generalized stable matching. They start with solving the relaxed version of the problem (assuming complete preferences), thereby potentially introducing instability, and then deleting unstable pairs through an iterative process until stable solutions are found.
- *Genetic Algorithm in combination with Threshold Acceptance*: [4, 33] suggest the use of Genetic Algorithms (GA) for the general problem with incomplete preferences and ties. The GA is initialized with a set of 50 different (but stable) starting solutions, which are calculated by arbitrary tie breaking and using the DA to calculate a stable solution. These starting solutions are then evolved by using mutation and crossover operators. Crossover operators take two existing stable solutions, exchange certain (randomly selected) parts of the respective solution, and uses the resulting new solutions in the next evolution step. Mutation operators randomly switch participants in two matched pairs, resulting in local changes to the solution. The GA typically uses the best solution of the population after 100 evolution rounds. After this initial GA, a Threshold Accepting (TA) algorithm is used to further improve the solution quality. The TA evaluates small adjustments to the solution (similar to the Mutation in GA) in every round until no improvement can be found. TAs are efficient in finding local improvements, as shown in [33], and well suited to complement the GA. We use a variant of the heuristic for the evaluation, *GATA-Mixed*, which uses a mix of DA, Király, McDermid, GSModified, and LocalSearchSMTI starting solutions for the GA phase. This particular method has been shown to perform well in similar settings [7].

### Optimal solutions

Conceptually, we can also formulate the problem of finding maximum sized matchings as constraint optimization problem. Using a formulation similar to [34] and [35], the SMTI one-to-one matching problem can be formulated as follows. The difference in this formulation is in Eq (5), which stems from a slightly different definition of the ≿ relation. Conceptually, however, the two formulations are equivalent:

$$\begin{array}{c} max_{\left(x,y\right)}\;\sum_{i\in X,j\in Y}z_{i,j} \end{array}$$ (2)

$$\begin{array}{cc} \sum_{i\in X}z_{i,y}\leq 1 & \;\forall y\in Y \end{array}$$ (3)

$$\begin{array}{cc} \sum_{j\in Y}z_{x,j}\leq 1 & \;\forall x\in X \end{array}$$ (4)

$$\begin{array}{cc} \sum_{j\;{≿}_{x}\;y}z_{x,j}+\sum_{i\;{≿}_{y}\;x}z_{i,y}+z_{x,y}\geq 1 & \;\forall \langle x,y\rangle \in A \end{array}$$ (5)

$$\begin{array}{cc} z_{x,y}\in \{0,1\} & \;\forall x\in X,y\in Y \end{array}$$ (6)

$$\begin{array}{cc} z_{x,y}=0 & \;\forall (x,y)\in (X\times Y)\backslash A \end{array}$$ (7)

Note that this optimization problem is NP-hard, and thus only tractable for a small number of participants. For our three matching scenarios, we can calculate the optimal solution and use it as a baseline reference against which we can compare the other algorithms. As IBM CPLEX was used to calculate the optimal (maximum cardinality) solution, the ‘CPLEX’ identifier will be used to indicate this particular solution.

### Manipulation strategies

In centralized Two-Sided Matching mechanisms, participants submit their preferences to a central instance (e.g., clearinghouse with a specific solution algorithm), which then calculates a solution based on the information submitted by the participants. In general, the question whether participants will submit their actual preferences or manipulations thereof depends on the incentive compatibility of the underlying mechanism. If it is not in their best interest, some participants might submit untruthful information, i.e., information that does not reflect the true preferences of the participant. While using manipulated preferences might be beneficial to individual participants, it leads to several detrimental effects for the entire system. First, the calculated allocation might not be the best overall solution in case all participants would disclose their true preferences. Second, preference manipulation can also lead to the emergence of blocking pairs with respect to the true preferences. More specifically, if the mechanism assumes that all submitted preferences are truthful and the resulting match based on these submitted preferences is stable, some participants might have incentives to deviate from the solution if they can switch with other participants and be better of (i.e., if they can find blocking pairs).

In order to study the implications of a lack of incentive compatibility for the existing mechanisms, the actual manipulation strategies need to be defined. Previous work has considered a set of different preference manipulation strategies, which are introduced subsequently.

#### Truncation

Conceptually, listing more participants of the opposite site as acceptable in one’s preference profile might increase the chances of being matched to one of the less preferred options. Hence, deciding which participants to include in the profile is an important strategic consideration. Artificially decreasing the number of acceptable participants can thus potentially increase the chances of being matched to a more preferred alternative. However, stating otherwise acceptable alternatives as unmatchable also increases the chance of the participant being unmatched, as it decreases the options that the mechanism can consider. This consideration describes the *truncation* strategy defined by [12]. Given the true preference ranking of length *n* of participant *i*, a truncation is defined as the preference ranking that contains the first *k* participants, *k* < *n*, in the same order as the true preferences. [12] showed that truncation strategies dominate non-truncation strategies under certain assumptions for the preference rankings, making them an interesting candidate for the following evaluation.

Truncation strategies involve an inherent trade-off. Truncating to a high degree aims to avoid being matched to less preferred alternatives, yet simultaneously increases the probability of remaining unmatched. [12] showed that for a given preference set, the number of participants benefiting from truncation when using the DA mechanism is small, yet its behavior under other algorithms or indifferences in preference rankings remains to be explored. Using analytical models, [36] extends the analysis of [12] for priority-based and linear programming mechanisms, and shows that under certain assumptions (symmetric information) the same result about truncation preferences holds.

#### Re-ordering

In general, stable solutions calculated by matching algorithms cannot guarantee that each participant is matched to its most preferred option. Therefore, it is likely that participants will be matched with their 2nd, 3rd, 4th, etc. choice, depending on the specific outcome. One potential strategy for participants who suspect that they are not likely to be matched with their most preferred alternative is shuffling or re-ordering: Putting more preferred alternatives (in their true preferences) in lower ranks might result in a better match for them. For example, if a participant is on average matched to its third choice, putting their true first choice at rank 3 might yield a better result for them. However, as the matching depends on the preferences of the other participants, it is not straightforward to see whether such a strategy might be useful. Furthermore, for strict and complete preferences [11] show that not putting the most preferred alternative first is a dominated strategy for the Deferred Acceptance Algorithm.

The re-ordering or shuffle strategy thus creates a new preference ranking which is submitted to the mechanism. It can be described by the degree of manipulation *k*, which defines that the strategy randomly shuffles the first *k* ranks. We will consider different degrees of manipulation in our subsequent evaluation and study its effects on the matching outcome.

#### Strategic re-ordering

While the previous re-ordering strategy is a potential option to manipulate preferences, a more strategic version of re-ordering should include a user’s perceived chances of getting matched to their preferred choice into account. For example, if a user knows that a particular mentor is very popular, they could put another mentor as preferred choice due to the low likelihood of getting matched to the actual preferred choice (while still wanting to get matched to a good option). A similar type of strategy was observed in the Boston school choice mechanism, where the popularity of a school influenced the likelihood of participants to include additional ‘safe choices’ in their preferences [37]. Hence, we also consider a more strategic version of re-ordering: For this, the user first estimates the popularity of a choice using the number of times the choice appears in the first *k* preference ranks of the other users. Then, the user calculates a relative popularity by multiplying this popularity with the preference rank of the particular user in one’s preference rank. Finally, the user selects the option with the lowest relative popularity score and puts that option as new choice for preference rank 1.

To illustrate this strategy, consider following example: A user has a preference ranking 1 ≻ 2 ≻ 3. Based on all users, user 1 has a popularity of 4, user 2 a popularity of 3, and user 3 a popularity of 1. The relative popularity scores would be [4 * 1, 3 * 2, 1 *3] = [4, 6, 3] users 1, 2, and 3, respectively. Hence, picking the smallest score, the user would put option 3 as new preferred rank.

The reason behind this strategy is straightforward: the user wants to maximize the combination of being matched to a good choice, while taking into account the likelihood of being matched to said choice. In realistic applications, the challenge of this strategy would be to get realistic estimates of the popularity score. However, in a mentor-mentee setting, such information could be available through perceived interactions with certain mentors / mentees at common workshops and meetings.

### Preferences

For the preferences, we used a set of real preferences coming from a mentor-mentee matching at our university. In total we have three instances of mentor-mentee matching preferences stemming from the last three years of the program. After an initial meeting, mentees provided a preference ranking for mentors based on common (research) interests and other considerations. Table 1 provides an overview of the preferences and their structure. For this, preferences that were not mutually acceptable were deleted in the respective preference rankings, leading to the deletion of a small number of mentors/mentees for the scenarios. The first set (subsequently referred to as scenario) consists of 20 mentees and 22 mentors, the second set of 23 mentees and 24 mentors, and the third set of 28 mentees and 33 mentors who wanted to participate in a college-wide program. As we can see, the third scenario involved larger preferences (i.e., more acceptable preferences), as well as a considerably larger number of preferences with ties.

**Table 1:** Preference overview.

Scenario	Mentors	Mentees	Avg Length Mentor Preferences	Avg Length Mentee Preferences	Percentage Preferences with Ties
1	22	20	7.14	3.6	0.54
2	24	23	23.0	4.87	0.51
3	33	28	25.2	5.96	0.97

## Evaluation: Solution quality

The first part of the evaluation considers the relative performance of the algorithms with respect to three main metrics used to quantify the solution quality of Two-Sided Matching solutions: Number of users matched, welfare, and fairness. We ran several simulations as described in the subsequent sections and evaluated the resulting outcomes.

### Simulation parameters

To evaluate the performance of the various algorithms with respect to finding high-quality solutions, a simulation-based evaluation approach is used. Specifically, several algorithms require a tie-breaking to calculate their solutions, and the (usually random) way of breaking these ties can influence the resulting solutions. Hence, we independently repeat all scenarios 100 times and report the averaged results for the respective algorithms. 100 repetitions are a commonly selected number of simulations to balance simulation run time and the ability to analyze algorithms and strategies in different scenarios (different preferences in our case), while also avoiding to potentially declare overly small effect sizes as significant (see e.g. [38]).

The simulation parameters used for the evaluation are shown in Table 2. We consider real preferences obtained from three years of matching Mentors to Mentees, as well as several solution algorithms. The optimal solution refers to the solution obtained through a CPLEX implementation of the optimization problem given in Eq 2, and by definition corresponds to the solution that yields the largest number of matched users for a given scenario (yet is only solvable for smaller instances). Additionally, the performance on several standard evaluation metrics is reported and analyzed.

**Table 2:** Simulation input parameters for comparing algorithm performance.

Parameter	Range	Description
*n*_*X*_	{20, 23, 29}	Number of mentees
*n*_*Y*_	{22, 24, 33}	Number of mentors
*algo*	{*DA*, *Kiraly*, *GSM*, *McDermid*, *GATA*, *CPLEX*}	Solution Algorithm
*sc*	{Matching Size, Welfare, Fairness, Stability}	Solution Criteria

### Finding maximum sized matchings

The prime goal of practically all established solution approaches in Two-Sided Matching is to find a solution of maximum size. As discussed before, for the number of participants in our case study we can calculate the optimal solution (with respect to number of participants matched) and compare the approaches against each other.

#### Number of participants matched

Starting with the main objective in current approximation algorithms, we compare the performance of the different approaches in the case where all participants submit their preferences truthfully (specifically, we assume that the submitted preferences are the true, non-manipulated preferences).

**Fig 1:**
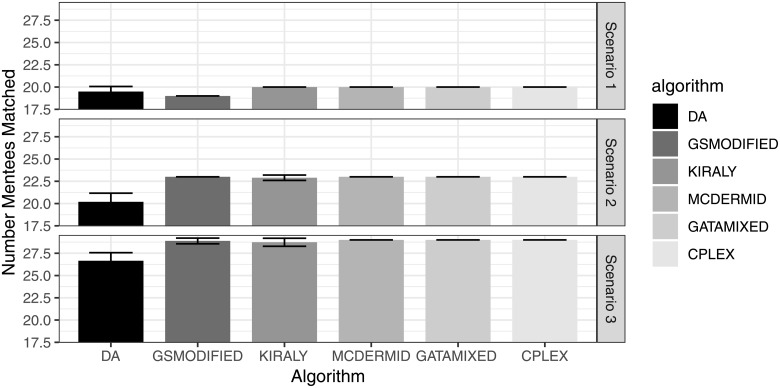
Absolute number of matched participants.

**Fig 2:**
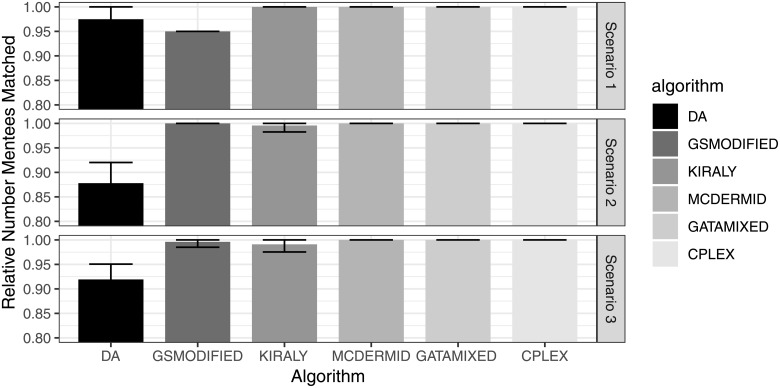
Relative number of matched participants compared to optimal solution.

Figs 1 and 2 show the absolute number of matched participants and the relative number of matched participants compared to the optimal solution, respectively. Overall, the average number of matched participants is very similar across algorithms, with MCDermid and GATA-Mixed finding the maximum number of matched participants in all of the 100 repetitions for both scenarios (Kiraly finds the maximum number in the first scenario, but not always in the second or third scenario). Also, both GSModified and the original DA algorithm have slightly lower performance, with the DA averaging to find the maximum sized solution in 87.8%—97.5% of the cases, and GSModified in 95.1%—100% of the cases. The differences seem to me larger for the second scenario with more participants, which is not surprising as the increased number of participants increases the complexity of the overall problem.

In addition to the number of matched participants, we now consider the performance of the algorithms with respect to Welfare and Fairness. Recall that Welfare indicates how close to the most preferred solution the average participant is matched with, and Fairness compares the two Welfare scores for mentors and mentees. In this sense, a Welfare score of 1 indicates that the average participant is matched to their most preferred option, whereas a Fairness score of 0 indicates that both sides are treated perfectly fair.

**Fig 3:**
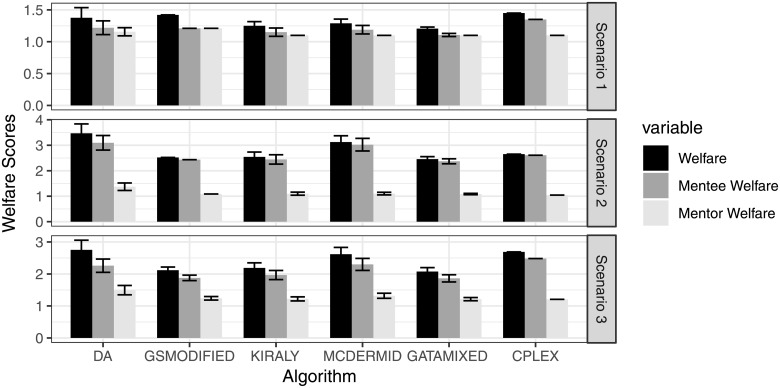
Average welfare scores for participants.

Fig 3 shows the average Welfare for the algorithms, as well as the average Mentor Welfare and Mentee Welfare. We can see that the average overall Welfare scores across algorithms are fairly similar, yet we do observe certain differences. First, GATA-Mixed seems to yield the best quality with respect to Welfare. On average, it achieves Welfare score of 1.21, 2.45, and 2.08, as compared to the second best solutions Kiraly and GSModified with scores of 1.25, 2.54, and 2.19, as well as 1.42, 2.52, and 2.18 respectively (a reduction between 3% and 14%). Considering mentee Welfare, GATA-Mixed again provides the best results, outperforming the second best solutions by 1% to 6%. The error bars (plus and minus one standard deviation of the results) indicate that there is some fluctuation across the 100 repetitions, yet the general ranking of the algorithms seems clear. Considering Welfare differences between mentors and mentees, we see that the average welfare of mentors seems to be smaller than that of mentees. Specifically, mentors are matched close to their first choice on average, while mentees are matched to their second choice on average (third, depending on the algorithm). This is not surprising, however, when the structure of the preferences is taken into consideration. In the three scenarios, mentors have preferences with considerably more indifferences, meaning that they are happy with the match as long as the mentee is acceptable for them. In contrast, mentee preferences include a higher percentage of strict preferences, which makes it less likely for them to always be matched to their first choice. Differences between algorithms are similarly visible when we break down overall Welfare into Mentor and Mentee Welfare, where GATA-Mixed seems to outperform the other algorithm.

**Fig 4:**
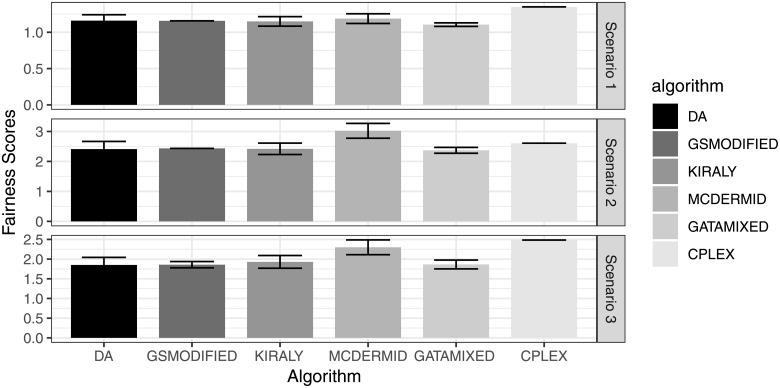
Average fairness score of solutions.

Finally, Fig 4 shows the Fairness scores of the different algorithms. On the one hand, we see that the performance of the algorithms is fairly similar again, despite minor fluctuations. GATA-Mixed seems to perform best on average, even though the error bars show that other algorithms (e.g., DA in this case) sometimes lead to equally good or better results.

Summarizing the comparison of the algorithms with respect to the three performance metrics, we saw that for the given scenarios, McDermid and GATA-Mixed always yielded the maximum number of matched pairs. In addition, GATA-Mixed seems to consistently outperform the other approaches with respect to secondary metrics such as Welfare and Fairness. This can be attributed to the fact that GATA-Mixed is specified as a multi-objective heuristic, whereas the other algorithms are approximation algorithms focusing on the number of matched pairs. Overall, for the given scenarios, GATA-Mixed seems to be the preferred option as it yields solutions that are favorable for the overall system (matching as many participants as possible, thus maximizing the number of mentor-mentee pairs), and for the participants (matching them as close to their most preferred choice as possible).

## Evaluation: Preference manipulation

### Simulation parameters

For the preference manipulation simulation, we again consider the previously discussed data sets. The parameters used for this part of the evaluation are shown in Table 3. For a given data set, we vary the percentage of manipulating participants *p*_*M*_ and the type or degree of manipulation *d*_*M*_. The manipulation type can be truncation, re-ordering/shuffling, or strategic re-ordering/shuffling (see the previous sections for details). For truncation strategies, the degree of manipulation defines how much the preferences are truncated. For example, in a scenario where non-truncated preferences have a length of 10, truncation of degree 0.5 means that the truncated preferences have length 5. To ensure the robustness of the findings, in particular accounting for the fact that many algorithms employ random elements and thus potentially yield different solutions at every run, 100 independent repetitions are made for each scenario. The results are averaged over these 100 runs.

**Table 3:** Simulation input parameters for the manipulation study.

Parameter	Range	Description
*n*_*X*_	{20, 24, 29}	Number of mentees
*n*_*Y*_	{22, 23, 33}	Number of mentors
*p*_*M*_	{0, 0.1, 0.2, …, 0.9, 1}	Percentage of manipulating users
*s*	{shuffle, strategic shuffle, truncation}	Manipulation strategies
*d*_*M*_	{0.1, 0.2, …, 0.9}	Degree of manipulation

In general, both mentors and mentees can manipulate their preferences, i.e., both sides can potentially manipulate. In the case of the DA algorithm and the shuffle and strategic shuffle strategies, however, we only consider manipulation by the mentors, i.e., one-sided manipulation. This is based on the theoretical analysis by who show that putting the most preferred option first is a weakly dominant strategy for the proposing side in the DA algorithm. That is, users on the proposing side (see [13] for details) should not use shuffling strategies from a rational point of view.

### Effects of manipulation on participant outcomes

The inherent goal of participants who manipulate their preferences is to improve their outcome, i.e., to be matched to a more preferred option. Hence, we start our analysis with evaluating the effects on the manipulating participants’ outcome. As we consider two parameters that affect manipulation, degree of manipulation and percentage of manipulating users, we evaluate these effects separately (by averaging over the other parameters).

**Fig 5:**
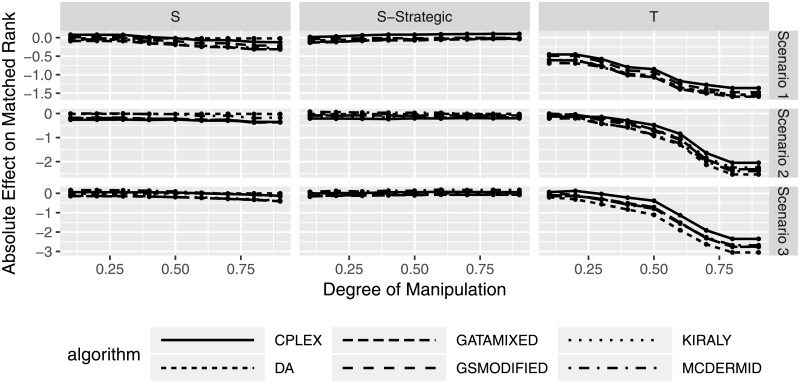
Change in matched rank for manipulating participants based on degree of manipulation.

Fig 5 shows the absolute effect of manipulation for the manipulating users based on a change in their matched rank, given different degrees of manipulation. The figure shows several interesting results. First, on average both the truncation and the shuffle strategies lead to a decrease in matched rank, whereas the strategic shuffle strategy leads to absolute effects fluctuating around 0. This means that effectively, manipulating participants are mostly worse off (on average) than if they would not manipulate, or in case of the strategic shuffling, the average result would not change considerably. Second, the shuffle strategies lead to less extreme changes in average matched rank, whereas truncation to a high degree can lead to substantial losses in welfare (matched rank) for the manipulating participants. Third, the average loss in matched rank increases with increasing (more aggressive) manipulation for truncation strategies, indicating that manipulating participants can quickly be worse off by this strategy. Fourth, we see that in some cases, the change average matched rank is positive, indicating that manipulation can slightly pay off in these cases. However, compared to the overall behavior, manipulation on average still leads to a loss. Finally, we see that there is not much difference between the solution algorithms, indicating that the observed behavior is a result of the manipulation itself and not an artifact from a particular solution procedure.

**Fig 6:**
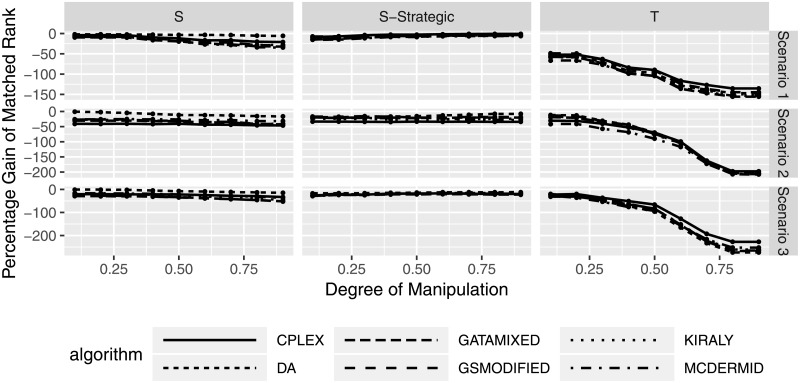
Relative change in matched rank for manipulating participants based on degree of manipulation.

In addition to the absolute change in matched rank, Fig 6 shows the relative change in matched rank. This is calculated as the percentage change of manipulating participants’ matched rank using the respective rank in the non-manipulated case as baseline. A value of -100 would indicate a relative loss of 100% compared to the previous matched rank. We can see that, similar to the absolute matched rank comparison, truncation leads to considerable relative losses in matched rank, especially for higher degrees of manipulation. In the case of shuffling strategies, relative improvements and losses again fluctuate around 0%, with strategic shuffling leading to fewer differences between the algorithms.

**Fig 7:**
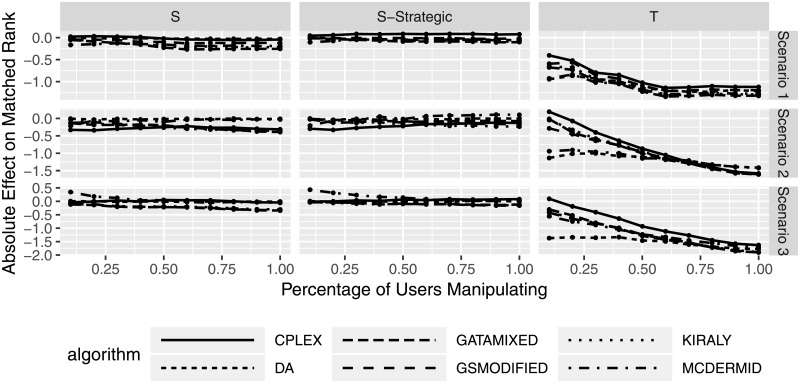
Change in matched rank for manipulating participants based on percentage of manipulating participants.

**Fig 8:**
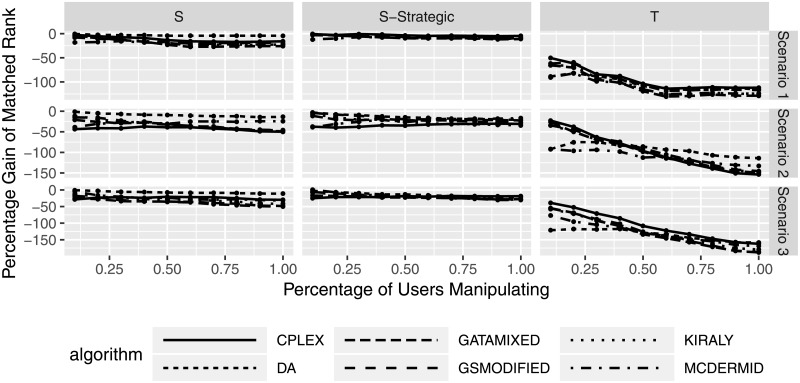
Relative change in matched rank for manipulating participants based on percentage of manipulating participants.

The previous considerations considered different manipulation degrees, i.e., how severely the preferences were manipulated (shuffled or truncated). Figs 7 and 8 consider the effects of the percentage of manipulating participants, i.e., how many participants actively submit manipulated preferences. Overall, the results are quite similar to the previous evaluation. Manipulation using the shuffle strategies seem to lead to less gains or losses than truncation strategies, and the effect of manipulation seem to increase (both positive and negative) with an increasing number of manipulating users. With the lack of difference between solution algorithms, we can see that neither shuffle strategy seems to be a particularly promising manipulation strategy for participants. On the other hand, a higher number of manipulating users leads to a loss in average matched rank (welfare) of manipulating participants. In other words, the more participants try to improve their outcome by misstating their preferences, the worse off they will be (on average). Algorithm behavior for truncation is also quite similar, with certain algorithms leading to smaller or higher average differences in matched rank.

Fig 8 shows a more pronounced differentiation between solution algorithms, even though the absolute differences between the approaches are quite small.

**Fig 9:**
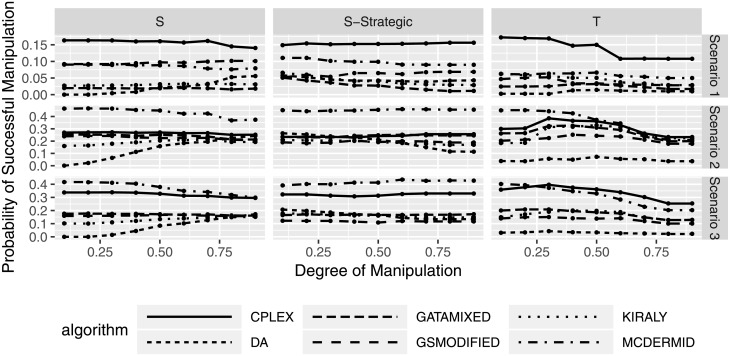
Probability of successful manipulation based on degree of manipulation.

**Fig 10:**
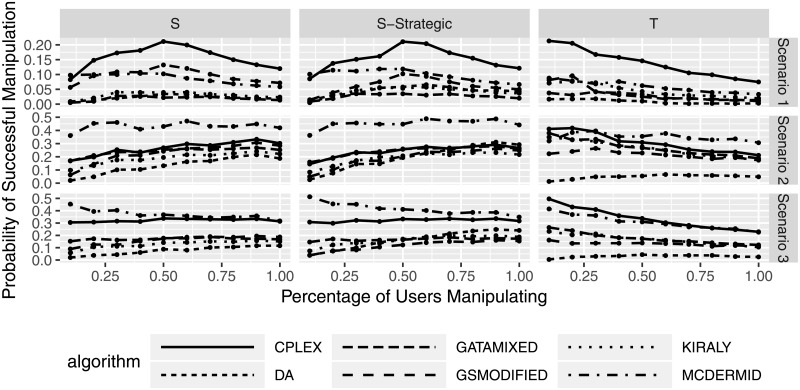
Probability of successful manipulation based on percentage of manipulating participants.

While the previous figures show that manipulation only seems to be beneficial in a small subset of cases while leading to average losses, we will now look at an manipulating individual’s probability that its manipulation is successful (in the sense that the average matched rank gets better). Figs 9 and 10 show the probability that submitting manipulated preferences lead to a better outcomes for the manipulators. Success in this case is defined as the percentage of simulation scenarios in which submitting the manipulated preferences leads to an improvement. The figures show that success probabilities are mostly between 0% and 20% in the first scenario, between 0% and 50% in the second scenario, and between 0% and 45% in the third scenario. While this might seen promising, the interpretation of this is that in 80%, 50%, and 55% of the cases in scenario 1, 2, and 3, respectively, manipulation does not pay off. In addition to the average losses from manipulation as seen earlier, this leads us to conclude that the potential gains of manipulation seem to be outweighed by the potential losses, at least for the preferences studied here. Interestingly, Fig 10 shows that the probability of successful manipulation first increases, then decreases again for shuffling strategies with an increasing number of manipulating users, while truncation strategies seem to be less successful overall when many users manipulate. An interpretation of this result is that many manipulating users introduce complexity into the system that makes it less likely to benefit from active manipulation.

Finally, we see that there seems to be a difference between solution algorithms. In particular, CPLEX and McDermid seem to have the highest probability of success, whereas DA and GATA-Mixed seem to have the smallest success probabilities. This result needs to be carefully considered along the algorithm performance results in Section to select a solution algorithm with favorable properties both for solution quality and susceptibility to manipulation.

### Effects of manipulation on non-manipulating participants

The previous section considered the effects of manipulation and success probabilities for the manipulated participants. However, there is a second side that needs to be investigated: the effects of manipulation on truthful, non-manipulating participants. In particular, we want to investigate to what degree these participants are affected by the presence of manipulation in the matching.

**Fig 11:**
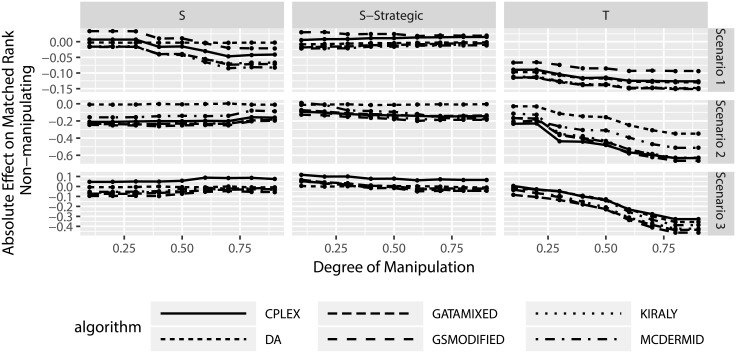
Change in matched rank for non-manipulating participants based on degree of manipulation.

**Fig 12:**
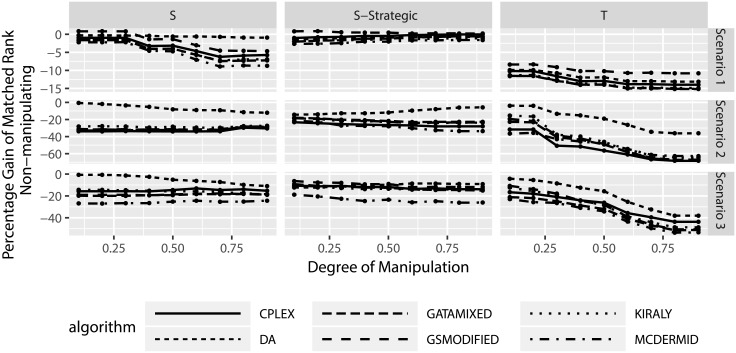
Relative change in matched rank for non-manipulating participants based on degree of manipulation.

Figs 11 and 12 look at the absolute and relative change in matched ranks, this time for the non-manipulating participants. Several aspects can be observed from the figures. First, on average non-manipulating users are worse off when other users manipulate. This is true both in the absolute change in matched ranks as well as the relative change in matched ranks. In particular the relative change in matched ranks indicates that non-manipulating participants can be considerably worse off. Second, the truncation strategy leads to worse outcomes for non-manipulating participants than the shuffle strategies. Third, there seems to be little difference between the solution algorithms, with the exception of the truncation strategy in scenarios 2 and 3, where we observe that three algorithms lead to smaller losses for non-manipulating participants (DA, GSMODIFIED, and MCDERMID).

**Fig 13:**
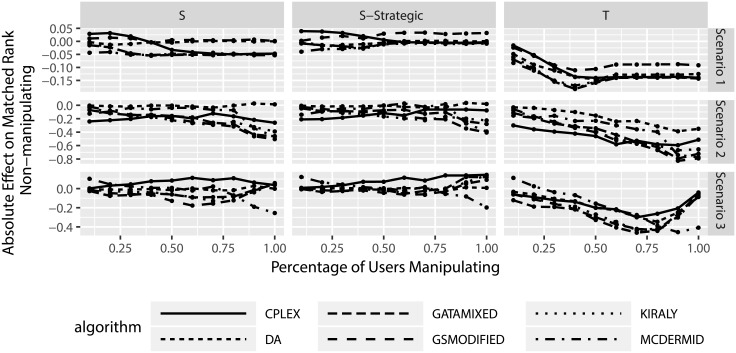
Change in matched rank for non-manipulating participants based on percentage of manipulating participants.

**Fig 14:**
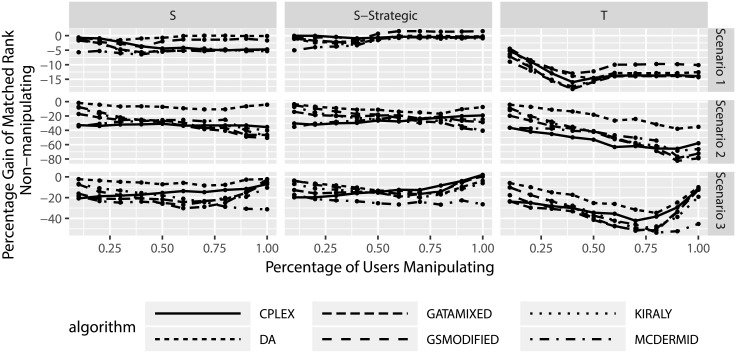
Relative change in matched rank for non-manipulating participants based on percentage of manipulating participants.

Similar to before, we also consider the effect of the percentage of manipulating users on the results. Figs 13 and 14 show several interesting results. First, increasing the number of manipulating participants seems to increase the losses for non-manipulating participants. This indicates that the more manipulation happens in the Two-Sided Matching, the worse off the non-manipulating participants tend to be. Second, for truncation there seems to be a reverse in this trend in scenario 3, where manipulation suddenly leads to less losses for non-manipulating participants when nearly all other participants manipulate. However, due to the fact that in this case only single participants do no manipulate and the sample size of this consideration is reduced, this particular result can be a statistical artifact and should not be over-emphasized.

### Effects of manipulation on stability of solution

Having evaluated the effects of manipulation both on the manipulating as well as the non-manipulating participants, we now consider manipulation effects on the overall Two-Sided Matching market. As discussed previously, stability is the major goal of any solution in Two-Sided Matching and thus is enforced by all solution algorithms. However, the stability that the solution algorithms calculate is based on the submitted preferences, not the truthful preferences that participants might keep for themselves. Hence, it is entirely possible that solutions will be stable under the submitted preferences, yet not under the actual preferences.

**Fig 15:**
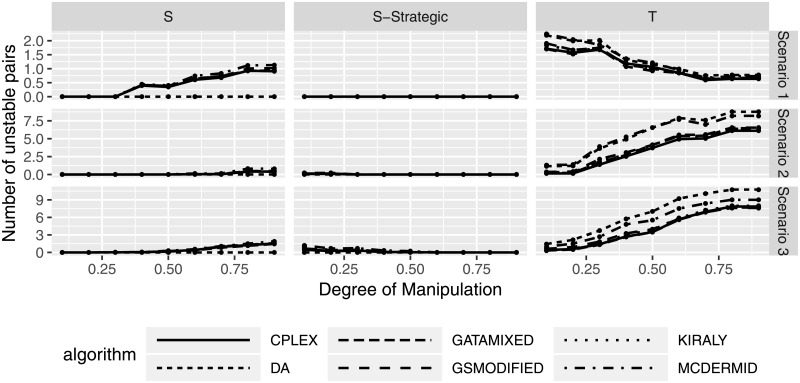
Number of unstable pairs based on degree of manipulation.

**Fig 16:**
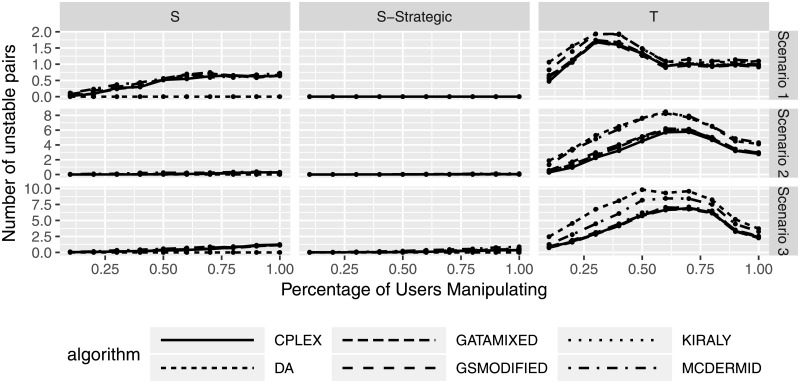
Number of unstable pairs based on percentage of manipulating participants.

To study this potentially hazardous effect, we compare the solutions calculated based on the submitted, manipulated preferences to the original, truthful preferences. I.e., as we know the actual preferences in our simulation setting, we can calculate the number of unstable pairs that solutions calculated based on manipulated preferences have. Figs 15 and 16 show the number of unstable pairs based on different manipulation degrees and percentage of manipulating participants. We can see that the average number of unstable pairs, based on the original preferences, seems to be between 0 and 2 in scenario 1, 0 and 5 in scenario 2, and between 0 and 10 in scenario 3.

Interestingly, while both shuffling strategies and truncation can lead to instability in many cases, strategic shuffling seems to lead to fewer unstable pairs, with instability occurring for small degrees of manipulation (i.e., only considering the top choices for a switch) or a high percentage of manipulating users. A potential explanation for this type of observed behavior is that this type of strategy does not considerably affect the resulting matching, i.e., some users changing their first preference rank does not seem to impact the resulting (stable) solution. While these numbers do not seem to be particularly large, they do indicate that instability can be introduced by preference manipulation. In particular with the knowledge that even one unstable pair can lead to a complete unraveling of the entire solution, this is a result that needs to be emphasized, as it shows that manipulation can lead to potentially significant effects on the overall solution (and the acceptance of the solution) in Two-Sided Matching.

## Discussion

The previous evaluation showed several interesting results with respect to differences in solution algorithms as well as effects of preference manipulation.

Considering Research Question 1, the solution quality for the three studied scenarios, we saw that several algorithms were able to match the optimal algorithm for the number of participants matched, i.e., the size of the matching. In addition, all the considered algorithms were reasonably close to the maximum size matching. As this is the most common optimization target (acknowledging that stability is implicitly assumed), the results are encouraging, in particular for applications with a smaller number of participants. Furthermore, when we take secondary metrics such as Welfare (average matched rank) and Fairness (Welfare differences of the two sides) into account, we saw that the algorithm GATA-Mixed provided strong results by yielding better Welfare scores while retaining the maximum size matching. Overall, for the given preferences, mentors were matched closer to their best choice than mentees. For participants of this matching, this is an added benefit as it ensures that many mentor mentee pairs can be established, and at the same time the mentors and mentees are on average matched close to their most preferred option. Hence, even in scenarios where the optimal number of matched participants can be calculated, it is worthwhile to consider other approaches such as multi-objective heuristics, as they might be able to improve upon other metrics.

To answer the second research question, we studied several effects of preference manipulation on participants and the overall solution quality. The summary of the results are:

- Considering the three manipulation strategies, shuffling, strategic shuffling, and truncation, the results showed that the truncation strategy leads to larger effects, i.e., the gains and losses from manipulation are considerably larger.
- On average, manipulation leads to losses for both manipulating and non-manipulating participants. Whereas there are cases where manipulation can lead to small gains, the majority of the evaluated cases showed a substantial loss from manipulation.
- Non-manipulating participants are practically always worse off when others submit manipulated preferences.
- In most cases, gains and losses seem to be independent of the solution algorithm that is used to calculate the solution. For certain algorithms the probability of a successful manipulation is different from the other approaches. For example, the success probability seems to be highest using the optimal CPLEX algorithm, and lowest for the DA and GATA-Mixed algorithms.
- Manipulation introduces instability into the final solution. The results showed that the solutions calculated based on manipulated preferences are not stable under the original preferences. This is a potentially dangerous side-effect of manipulation, as it is well known that even a single unstable pair can lead to an unraveling of the provided solution.

Overall, this study provided relevant insights into using different Two-Sided Matching algorithms, and in the usefulness (and danger) of submitting manipulated preferences. Given that the average benefit from manipulation was negative for our considered scenarios, there seems to be little practical incentives for participants to actually submit manipulated preferences.

## Conclusion

Two-Sided Matching is an established and well-suited approach to match participants based on their preferences. Applied in a variety of settings, various solution algorithms have been developed and target specific metrics for solution quality. In this article, we extend previous research in this area by providing a systematic comparison of several solution algorithms using realistic sets of preferences derived from a Mentor-Mentee application at our university.

Comparing the quality of the calculated solutions between the considered algorithms, we find that several algorithms are able to consistently find the ‘best’ solution with respect to stability and number of matched participants. Additionally, a considered multi-objective heuristic was able to improve secondary objectives such as the average matched rank and fairness of the solution, while retaining good performance on the other metrics.

Considering the effects of preference manipulation, we studied three types of potential manipulation (truncation, shuffle, and strategic shuffle) over a variety of scenarios. The results for the given set of preferences show that manipulation is rarely beneficial and on average leads to potential losses for the manipulating participants. Additionally, we find that manipulation by participants introduces instability in the solution, which is a potentially hazardous result as the effects of instability can cause the entire solution to unravel.

We believe the results of this evaluation provide relevant guidance and insight into the different solution algorithms and the effects of preference manipulation. Going forward, there are several aspects that we plan to consider. First, we want to extend the analysis to include a broader set of preferences. This will allow us to get more generalizable results and study the effect of certain model parameters on the observed outcomes. Second, we will consider more advanced forms of preference manipulation, for example through active learning techniques used by participants instead of passively selecting one single strategy. Third, we will consider the evaluation of cardinal utilities and their effect on the resulting Welfare and Fairness scores, taking into account that users might have strongly different preferences for their top choices but not highly different preferences for lower ranked choices. Finally, we want to explore how Two-Sided Matching can be applied for dynamic matching scenarios instead of one-time scenarios. This can open up an entire new area of applications for Two-Sided Matching and provides a fruitful area of investigation.

## Supporting information

S1 Data The preference sets used in this manuscript, in addition with summary tables of the results and a Data Dictionary providing information on the variables. The complete set of matching outcomes and manipulated preferences for each scenario and simulation run can be found in the repository https://zenodo.org/record/2555099 with doi 10.5281/zenodo.2555099.
(XLSX)
